# Tai chi in mental health interventions: a narrative review comparing its role with pharmacotherapy and psychotherapy

**DOI:** 10.3389/fpsyg.2026.1738619

**Published:** 2026-02-25

**Authors:** Xiaoqi Zhang, Jianqiao Wei, Tao Xiu

**Affiliations:** 1 College of Physical Education, Hebei Normal University, Shijiazhuang, China; 2 College of Physical Education and Health Sciences, Guangxi Science & Technology Normal University, Laibin, China; 3 College of Aviation Fundamentals, Naval Aeronautical University, Yantai, China

**Keywords:** cognitive behavioral therapy, integrated intervention, mental health, pharmacotherapy, TC

## Abstract

**Background:**

Mood disorders such as anxiety and depression represent a significant global disease burden. While pharmacotherapy (e.g., SSRIs) and Cognitive Behavioral Therapy (CBT) are mainstream interventions, they are associated with limitations including side effects, dependency, accessibility, and reliance on patient engagement. Mind–body exercises like Tai Chi (TC) have emerged as a potential complementary approach, but their comparative role and value within the intervention landscape remain to be clearly delineated.

**Objective:**

This narrative review aims to critically synthesize and interpret existing literature to compare the role of Tai Chi with pharmacotherapy and psychotherapy (primarily CBT) in mental health interventions. It seeks to elucidate TC’s potential benefits, limitations, mechanisms, and its integrative potential within a multimodal treatment framework.

**Methods:**

Employing a narrative review methodology, we conducted a purposive and critical analysis of key literature, including systematic reviews, meta-analyses, and pivotal randomized controlled trials identified through databases such as PubMed and Web of Science. The synthesis was guided by a conceptual comparative framework focusing on mechanisms, onset of action, applicability, side effects, and long-term outcomes. As a narrative review, this work prioritizes theoretical integration and interpretive analysis over systematic, exhaustive literature retrieval and quantitative synthesis.

**Findings:**

Our qualitative synthesis suggests that TC may offer a distinct, body-awareness-based intervention pathway. Compared to pharmacotherapy, TC appears devoid of drug-related side effects and may contribute to sustained wellbeing and overall health, albeit with a slower onset, making it potentially suitable as an adjunct in long-term management. Relative to CBT, TC provides a non-verbal, somatic approach that may complement cognitive restructuring by addressing physiological symptoms of anxiety and depression. Literature indicates that adjunctive use of TC alongside conventional treatments may yield synergistic benefits. However, evidence on long-term efficacy and optimal integration protocols remains preliminary, and findings are interpreted within the acknowledged limitations of heterogeneous primary studies. Hence, TC may hold value as a complementary mind–body intervention within mental health care. Its integration with pharmacotherapy or CBT seems promising but requires careful clinical structuring. Future research should prioritize high-quality trials on integrated protocols and the standardization of TC interventions. This review underscores the importance of a nuanced, patient-centered approach that considers TC as part of a broader therapeutic toolkit.

## 1 Introduction

Anxiety and depression, as prevalent mood disorders, contribute significantly to the global burden of disease, making the development of effective interventions a persistent priority in clinical and public health (Moore and Mattison, 2017). Current mainstream interventions primarily consist of pharmacotherapy and psychotherapy. Pharmacological treatments, such as Selective Serotonin Reuptake Inhibitors (SSRIs), are first-line therapies endorsed by clinical guidelines for their broad efficacy in alleviating symptoms via neurotransmitter regulation. Nevertheless, their use is commonly associated with side effects, including drowsiness, weight gain, and sexual dysfunction, alongside concerns regarding long-term dependency and elevated relapse risk following discontinuation (Bala et al., 2018; Montejo et al., 2015). Cognitive Behavioral Therapy (CBT), a representative form of psychotherapy with substantial empirical support, effectively assists patients in altering negative cognitions and maladaptive behaviors. However, its effectiveness relies heavily on patients’ cognitive engagement and therapists’ proficiency, limiting its accessibility for individuals with difficulties in verbal expression or those experiencing cognitive decline (Evans, 2007).

Against this background, mind–body interventions have garnered increasing attention as non-pharmacological, low-risk complementary and alternative approaches. Recent evidence underscores that structured physical activity and therapeutic exercise function as biologically active mental health interventions, exerting effects through mechanisms such as neurogenesis, neurotransmitter modulation, and stress-axis regulation (Kanani et al., 2025). Within this broader framework of “exercise as medicine,” traditional mind–body practices offer a structured approach that integrates physical movement with mental focus. Tai Chi (TC), originating from China, is one such practice that combines gentle, flowing physical movements, breath modulation, and meditative attention (Kanani et al., 2025). It represents a targeted, low-impact extension of exercise-based mental health strategies, with a growing body of literature exploring its specific potential (Kanani et al., 2025).

Preliminary research suggests TC may positively reduce symptoms of depression and anxiety and enhance overall wellbeing, with proposed mechanisms involving the enhancement of mind–body connection, improvement in mindful awareness, and facilitation of autonomic nervous system balance (Kong et al., 2019; Kishi et al., 2023). However, the precise role of TC within the broader landscape of mental health interventions remains inadequately delineated. Key questions persist: What are its specific comparative benefits and limitations relative to mainstream interventions? How does it compare with other mind–body practices like Yoga and Qigong? And can its integration with conventional therapies yield synergistic benefits?

To address these questions, this narrative review employs a multi-dimensional comparative framework (encompassing mechanisms of action, onset of effect, applicable populations, side effects/risks, long-term outcomes, and holistic health impact) to synthesize and interpret the existing literature. We aim to: (1) compare the benefits, limitations, and mechanisms of TC and pharmacotherapy; (2) delineate the differences and complementary potential between TC and CBT regarding intervention pathways; (3) elucidate the distinctive position and value of TC among other traditional mind–body exercises like Yoga and Qigong; and (4) investigate the potential advantages of integrating TC with other treatment modalities. By doing so, this review seeks to fill the current gap in integrative, cross-modal comparative synthesis, offering a nuanced perspective to inform clinical decision-making and guide future research directions in the application of Tai Chi for mental health.

## 2 Methodological approach

This work is a narrative review, which aims to provide a comprehensive, critical, and interpretative synthesis of existing literature on the role of Tai Chi (TC) in mental health interventions. Unlike systematic or scoping reviews, the primary objective is not to exhaustively catalogue all available evidence through a rigid, protocol-driven process. Instead, we seek to conceptually map the field, compare and contrast key intervention paradigms (pharmacotherapy, CBT, and other mind–body exercises), and develop a coherent theoretical narrative regarding TC’s unique positioning, mechanisms, and integrative potential. Our methodology emphasizes expert analysis, thematic integration, and the identification of overarching patterns and insights to inform clinical understanding and future research directions.

### 2.1 Literature identification and selection

Guided by the review’s comparative aims, we adopted an iterative and purposive strategy to identify relevant literature. The lead authors, whose expertise spans clinical psychology, exercise science, and integrative medicine, conducted targeted searches in major academic databases (PubMed, Web of Science, PsycINFO) using key term combinations (e.g., “Tai Chi” AND “mental health,” “Tai Chi” AND “depression/anxiety,” “Tai Chi” AND “pharmacotherapy/CBT,” alongside comparative terms for Yoga and Qigong). The search was focused on literature published between approximately 2010 and 2024, with seminal older works included for historical context.

The selection process was interpretive and conceptual. We prioritized identifying foundational and influential literature that could illuminate the core themes of our comparison. This included: High-impact evidence syntheses: Systematic reviews and meta-analyses that summarize the state of evidence for TC and comparator interventions. Pivotal clinical trials: Key randomized controlled trials (RCTs) that demonstrate efficacy, explore mechanisms, or involve direct comparisons. Theoretical and mechanistic studies: Articles proposing or testing explanatory models for mind–body interventions. Reviews on comparator modalities: Authoritative reviews detailing the benefits and limitations of pharmacotherapy and CBT, to ensure a balanced perspective. Inclusion was guided by the relevance to our pre-defined comparative dimensions (e.g., mechanism of action, efficacy, side effects, applicability). Rather than employing dual independent screening with rigid criteria, literature was discussed among the author team. Consensus was reached on its value in contributing to the conceptual framework, with a focus on methodological rigor, clarity of findings, and relevance to the comparative analysis.

### 2.2 Analytical and synthesis framework

The core of this narrative review lies in its analytical synthesis. After building a corpus of key literature, we employed a thematic comparative analysis. We extracted and organized information according to a matrix of conceptually derived dimensions critical for understanding mental health interventions: Mechanism of Action: The proposed biological, psychological, or combined pathways through which the intervention exerts its effects. Temporal Profile: Speed of onset and durability of effects. Applicability: Suitable patient populations based on symptom severity, cognitive capacity, and physical ability. Risk and Burden: Side effects, dependency risks, and demands on the patient (e.g., adherence, cognitive engagement). Holistic Impact: Effects beyond core psychiatric symptoms, particularly on physical health and quality of life. Synergistic Potential: Theoretical and empirical rationale for combining with other interventions.

Information from the selected literature was mapped onto this matrix separately for TC, pharmacotherapy, CBT, Yoga, and Qigong. This allowed for a structured side-by-side comparison, moving beyond mere description to identify contrasts, complementarities, and gaps. For instance, contrasting the rapid neurotransmitter modulation of pharmacotherapy with the gradual neurophysiological regulation attributed to TC created a clear differential profile. Furthermore, we engaged in interpretative synthesis to generate higher-order insights. This involved connecting findings across studies to propose explanatory models, such as how TC’s non-verbal, somatic approach might complement CBT’s cognitive focus within an integrated mind–body treatment model. Tables 1–3 serve as visual summaries of this comparative analysis, crystallizing the synthesized insights for the reader.

**Table 1:** Multidimensional comparative analysis of TC and pharmacotherapy in mental health intervention.

Comparative Dimension	Pharmacotherapy	TC	References
Mechanism of action	Directly modulates central neurotransmitters (e.g., serotonin, GABA, glutamate) to restore neurochemical balance and alleviate symptoms.	Activates the parasympathetic nervous system via mind–body integration (mindfulness cultivation + gentle movement), decreasing stress and facilitating emotional regulation capacity.	(Kanani et al., 2025; Vecera et al., 2023; Nuss, 2015; Qu et al., 2024)
Speed of onset	Rapid onset; agents, such as SSRIs can promote symptoms within the first week of treatment, suitable for urgent relief of distress.	Gradual onset; needs maintained practice (weeks to months), with impacts accumulating progressively over time.	(Taylor et al., 2006; Kuang et al., 2024)
Applicable population	Prioritized for severe cases (e.g., major depressive disorder, depression with psychotic features, high suicide risk).	Suitable for individuals with mild-to-moderate symptoms, those intolerant to medication, older adults; can be utilized as an adjunct to pharmacotherapy.	(Kong et al., 2019; Flint et al., 2019; Pompili and Goldblatt, 2012; Kuang et al., 2024)
Side impacts	Notable side impacts exist, such as drowsiness, weight gain, sexual dysfunction, and gastrointestinal discomfort.	No drug-associated side impacts; offers additional benefits, including improved physical flexibility and balance.	(Bala et al., 2018; Montejo et al., 2015; Kuang et al., 2024)
Long-term use risks	Risk of drug dependence (e.g., benzodiazepines) and tolerance development, commonly necessitating dosage modifications.	No known risk of chemical dependence; long-term practice is associated with improvements in psychological resilience and no evidence of tolerance.	(Moore and Mattison, 2017; Taylor-Piliae and Froelicher, 2004)
Relapse rate after discontinuation	High relapse rate; symptoms commonly reemerge after cessation, as treatment mainly delays symptoms without resolving underlying causes.	Some studies suggest sustained benefits; theoretical models and preliminary data indicate psychological skills may persist post-practice, potentially correlating with lower relapse rates in some populations.	(Care, 1998; Taylor-Piliae and Froelicher, 2004)
impact on overall health	Targets mental health symptoms specifically, generally lacking additional physical health benefits.	May promote both mental and physical health; may improve cardiovascular function, immune response, and sleep quality.	(Li et al., 2001; Ren et al., 2017; Chen et al., 2016)
Combined intervention impacts	Used alone or in combination with other medications, needing physician guidance for regimen modifications.	Combined use with pharmacotherapy can vitally improve drug effectiveness and possibly reduce dependency risks.	(Kong et al., 2019)

**Table 2:** Comparative and combined utilization analysis of TC and cognitive behavioral therapy (CBT) in mental health intervention.

Comparative dimension	Cognitive behavioral therapy (CBT)	TC	Potential advantages of combined application	References
Core mechanism of action	centers on cognitive restructuring; delays psychological symptoms by identifying/changing negative thought patterns combined with behavioral training (e.g., behavioral activation).	Centers on mind–body integration; activates the parasympathetic nervous system via slow movements + breath regulation + mindfulness practice, decreasing physiological tension and psychological stress.	Integrates “cognitive modification” and “somatic regulation,” intervening from both psychological and physiological dimensions to create synergistic impacts.	(Qu et al., 2024; Watkins et al., 2018; Liu et al., 2018)
Applicable population features	Suitable for patients with normal cognitive function who can actively take part in cognitive analysis; targeted for anxiety, depression, PTSD, etc.	Suitable for individuals with weaker cognitive abilities (e.g., older adults), those less adept at verbal expression, or patients with post-traumatic fear of verbal exposure; no strict age or physical fitness prerequisites.	Covers patients with varying cognitive levels and expressive abilities, allowing flexible modification of intervention focus based on individual needs.	(Evans, 2007; Chen et al., 2021; Laskosky et al., 2023)
Treatment duration and speed of onset	Short-term effectiveness, defined course (e.g., 12–20 sessions); widely cultivates coping skills via structured goal setting.	Gradual onset, needs long-term practice (weeks to months); impacts accumulate progressively over time, with more maintained long-term outcomes.	Combines “short-term skill acquisition” and “long-term mind–body adaptation”; CBT can offer wide symptom relief, while TC consolidates long-term impacts.	(Taylor et al., 2006; Ramnerö and Jansson, 2016; Liu et al., 2018)
Cognitive and participation demands	Requires high cognitive engagement, relies on patient self-reflection and verbal expression skills; needs adapted protocols for those with cognitive impairment.	Low cognitive threshold, centers on somatic awareness, does not need complex cognitive analysis; suitable for groups unable to take part in deep introspection.	Decreases the cognitive burden of CBT; TC helps patients build a sense of safety via physical practice, possibly improving engagement with CBT.	(Kanani et al., 2025; Qu et al., 2024)
Treatment process risks/limitations	Involves confronting negative emotions/trauma, which can trigger short-term emotional distress; effectiveness highly dependent on therapist expertise and skill.	Slower intervention speed for acute, severe psychological symptoms; needs long-term patient adherence, effectiveness susceptible to practice frequency.	TC’s mindfulness practice may help mitigate emotional distress triggered by CBT; CBT’s structured guidance can promote the focus of TC practice.	(Qu et al., 2024; Murray et al., 2022; Verhey et al., 2020)
Skill cultivation and transferability	Cultivates cognitive skills (e.g., cognitive restructuring, self-monitoring) transferable to controlling psychological distress in daily life.	Aims to cultivate somatic regulation skills (e.g., breath control, muscle relaxation), with added value of facilitating physical functions, such as balance and flexibility.	Forms a dual “cognitive-somatic” skill system, enabling individuals to both adjust thoughts and manage physiological reactions, improving overall adaptability.	(Li et al., 2001; Barton et al., 2023; Wang et al., 2023)
Intervention for specific symptoms	Well-established, evidence-encouraged first-line treatment for PTSD, notably via trauma-focused protocols.	Effective in decreasing physiological symptoms associated with anxiety/depression (e.g., muscle tension, wide breathing); non-verbal method may circumvent the need for direct verbal re-exposure to traumatic memories, potentially reducing distress.	For PTSD patients, CBT addresses traumatic memories while TC regulates physiological stress responses, possibly decreasing discomfort in the context of treatment.	(Kanani et al., 2025; Watkins et al., 2018; Laskosky et al., 2023)

**Table 3:** Clinically-relevant comparison of Tai Chi, Yoga, and Qigong.

Comparative dimension	Tai Chi (TC)	Yoga	Qigong	Clinical implication
Core movement pattern	Continuous, flowing motions; dynamic balance.	Static holds (asanas); stretching and strength.	Simple, repetitive or static poses; minimal motion.	*TC/Yoga:* improve coordination/flexibility. *Qigong:* lowest motor demand.
Typical intensity	Low-to-moderate (steady).	Highly variable (Gentle to Vigorous).	Very low (minimal exertion).	*TC/Qigong:* safest for frail/ill. *Yoga:* requires intensity matching.
Learning complexity	Moderate (linked forms).	Variable (posture alignment).	Low (simple repetitions).	*Qigong:* easiest start. *TC:* needs more instruction. Affects adherence.
Primary focus	Mind–body coordination in motion; balance.	Physical posture, breath, flexibility.	Breath-led mental focus and energy (“Qi”).	Guides choice based on patient goal (e.g., relaxation vs. body awareness).
Best-suited populations (examples)	Elderly, chronic disease, balance deficits.	Generally healthy, seeking flexibility/strength.	All, especially beginners, very frail, high-stress.	Population matching optimizes safety and benefit.

### 2.3 Positioning and limitations

We explicitly frame this as a narrative review to leverage its strengths in theory-building and conceptual clarity within a complex, multi-modal field. This approach allows for the integration of diverse types of evidence into a coherent argument. We acknowledge that the purposive, non-exhaustive search strategy may not capture every relevant study and is subject to author selection bias. However, this is counterbalanced by a deliberate focus on high-quality, formative sources to ensure the robustness of the comparative framework presented. The findings and conclusions are offered as a critical interpretation and synthesis of the current landscape, intended to clarify TC’s role, stimulate hypothesis generation, and guide more definitive future research, including rigorous systematic reviews and RCTs on integrated protocols.

## 3 Findings

### 3.1 Comparison between TC and pharmacotherapy

In the range of mental health intervention approaches, pharmacotherapy is a general method for treating mood disorders, such as anxiety and depression. While medications alleviate symptoms under the backdrop of regulating neurotransmitters (e.g., serotonin, dopamine), concerning their side impacts and long-term dependency remain focal points of research (Kishi et al., 2023). Conversely, TC, as a non-pharmacological intervention, displays potential in facilitating mental health (Kucukosmanoglu et al., 2024).

Evidence suggests that TC may alleviate symptoms of anxiety and depression and is associated with improved quality of life under the backdrop of impacting the mind–body connection (Wang et al., 2010). For example, literature from systematic review and meta-analysis has indicated that TC can decrease symptoms of depression and anxiety, as well as can increase patients’ overall physical and mental wellbeing (Kanani et al., 2025; Kong et al., 2019). Notably when integrated with pharmacotherapy, TC can vitally improve the therapeutic impacts of medication (Kong et al., 2019). However, the mechanisms of Tai Chi require further investigation to clarify its specific effects on neurotransmitters and emotional regulation (Kanani et al., 2025). Table 1 displays a multidimensional comparison between TC and pharmacotherapy in mental health intervention.

#### 3.1.1 Advantages of pharmacotherapy

##### 3.1.1.1 Wide symptom relief

Pharmacotherapy, notably antidepressants and anxiolytics, is generally utilized in clinical practice for the wide decrease of symptoms. Literature has reported that antidepressants, such as Selective Serotonin Reuptake Inhibitors (SSRIs) can begin to ameliorate symptoms under the backdrop of the first week of treatment (Taylor et al., 2006; Nakajima et al., 2010). For severely impacted individuals, these medications can quickly mitigate feelings of low mood and anxiety, aiding in the restoration of normal life functioning. Early pharmacological intervention in severe cases is positive in decreasing distress and improving quality of life (Taylor et al., 2006).

##### 3.1.1.2 Well-defined physiological mechanisms

Medications act by correcting imbalances in neurotransmitters within the central nervous system, a mechanism critical to a series of treatment protocols (Zhao et al., 2024). For example, GABA and glutamate are the main inhibitory and excitatory neurotransmitters, respectively, in the CNS, and their dysregulation is tightly associated with different neuropsychiatric disorders (Okada et al., 2023; Kaczmarski et al., 2023). In conditions, such as anxiety and depression, lowered GABAergic function or excessive glutamatergic activity can trigger symptoms (Vecera et al., 2023; Nuss, 2015). Under the backdrop of impacting the activity of these neurotransmitters, such as in the context of improving GABAA receptor function or decreasing NMDA receptors—pharmacotherapy can restore neurological balance and vitally alleviate symptoms (Lv et al., 2023). This mechanistic method underpins the action of numerous anxiolytic and antidepressant drugs (Nuss, 2015).

##### 3.1.1.3 Suitability for severe cases

Notably, for patients with severe major depressive disorder, severe anxiety disorders, or psychotic conditions, pharmacotherapy is commonly an indispensable component of treatment (Seifert et al., 2022). Literature has displayed that antidepressants and antipsychotic medications are positive in controlling these conditions and decreasing suicide risk. In cases of major depression with psychotic features, the integration of antidepressants and antipsychotics has reported positive in managing symptoms and avoiding relapse (Gregory et al., 2017; Flint et al., 2019). In addition, medications, such as olanzapine, clozapine, and certain mood stabilizers (e.g., lithium) show vital effectiveness in decreasing suicide risk (Pompili and Goldblatt, 2012). It is noteworthy that these drugs aid modulate patients’ mood and behavior, hence avoiding dangerous outcomes, such as suicide (Pompili and Goldblatt, 2012). Hence, for this type of patient populations, pharmacotherapy functions not only as a considerable tool for symptom control, but also as a core measure for avoiding disease development and safeguarding life.

#### 3.1.2 Disadvantages of pharmacotherapy

##### 3.1.2.1 Vital side impacts

Pharmacotherapy is commonly coupled with different side impacts, including drowsiness, weight gain, sexual dysfunction, and gastrointestinal discomfort (Zhou et al., 2023). It is noteworthy that these adverse impacts can negatively influence patients’ quality of life and may even cause treatment discontinuation in several individuals who find them intolerable (Zhou et al., 2023). Numerous general medications, such as antidepressants and antipsychotics, frequently drive these side impacts. For example, Selective Serotonin Reuptake Inhibitors (SSRIs) and antipsychotic drugs commonly trigger sexual dysfunction, including reduced libido or ejaculatory disorders (Bala et al., 2018). It is interesting that weight gain commonly results from drug-driven increased appetite or delayed metabolism, while gastrointestinal issues, such as nausea, constipation, or diarrhea are general short-term side impacts of antidepressants (Montejo et al., 2015). It is noteworthy that these side impacts can severely impact a patient’s quality of life, notably in the context of long-term treatment, possibly causing treatment abandonment. Hence, the clinical control of drug side impacts is critical (Montejo et al., 2015).

##### 3.1.2.2 Dependency and tolerance

Long-term use of certain antidepressants and anxiolytics can cause issues of drug dependency and tolerance. Literature has confirmed that antidepressants, such as SSRIs and benzodiazepines (e.g., diazepam-like drugs) can trigger dependence phenomena with prolonged utilization (Massabki and Abi-Jaoude, 2021). Once dependent, patients may need continuous utilization to sustain emotional stability, which can also precipitate withdrawal symptoms and enhance the risk of relapse upon discontinuation (Moore and Mattison, 2017). In addition, long-term utilization of these medications can cause tolerance, necessitating gradual dose improvements to sustain effectiveness, or even needing a switch to various drugs in several cases. This complicates the treatment regimen and is associated with a higher burden of side impacts and risks (Moore and Mattison, 2017). Hence, in the context of long-term pharmacotherapy for depression and anxiety, regular evaluation of therapeutic effectiveness and a careful weighing of the risks and advantageous of continued medication utilization are commonly suggested to avoid the trigger of dependency and tolerance issues.

##### 3.1.2.3 High relapse risk after discontinuation

The therapeutic impacts of pharmacotherapy are commonly difficult to maintain after drug cessation, and several patients are prone to relapse post-discontinuation (Bogers et al., 2020). This confirms that pharmacotherapy mainly delays symptoms instead of addressing the root trigger of the challenge, a phenomenon general in the treatment of drug dependency and mental disorders. Literature has reported that while pharmacotherapy is positive in the short term, long-term control remains a vital challenge, notably about relapse prevention (Zhu et al., 2018; Sihag et al., 2025). The relapse rate following pharmacological treatment is high, and symptoms commonly reemerge after discontinuation, revealing that symptom control alone is insufficient and stressing the necessity for integrative treatment and long-term control approaches (Care, 1998).

#### 3.1.3 Advantages of TC

##### 3.1.3.1 Absence of side impacts

As a non-pharmacological therapy, TC is extensively applicable to different populations, notably those intolerant to medications or sensitive to their side impacts (Lee and Chu, 2023). For instance, studies have reported that TC has vitally improved influences on anxiety and depressive symptoms in older adults, and unlike traditional pharmacotherapy, it does not generate drug-associated side impacts, making it suitable for individuals of various ages and physical conditions (Kuang et al., 2024). This low-intensity exercise form advantages both physical and mental health in the context of facilitating mood, flexibility, and balance (Kuang et al., 2024).

##### 3.1.3.2 Mind–body integration and holistic regulation

TC can not only modulate physical state, but can also help patients regulate emotions and promote psychological resilience via the cultivation of mindfulness and self-awareness (Chen et al., 2021). Article has revealed that TC, as a mindfulness-oriented physical activity, improves mindfulness, aiding practitioners center on the present moment, decrease stress, and facilitate emotional balance, hence exerting long-term effective influences on mental health (Chen et al., 2021). Particularly, mindfulness-improved TC (such as simplified 24-form TC) can positively decreasee symptoms of depression and anxiety and elevate mental health levels (Kanani et al., 2025; Qu et al., 2024). In addition, via its gentle physical movements and focused breathing exercises, TC can activate the parasympathetic nervous system, decreasing psychological stress (Qu et al., 2024). It is noteworthy that these features cause its vital interventional effectiveness in the range of mental health.

##### 3.1.3.3 Vital long-term impacts

A growing body of literature suggests the potential for long-term benefits from TC practice, particularly in enhancing self-efficacy and coping strategies (Tong et al., 2018). Some RCTs and systematic reviews indicate that TC may contribute to sustained improvements in psychological wellbeing (Taylor-Piliae and Froelicher, 2004). Theoretically, and supported by some observational data, the skills learned through TC (e.g., mindfulness, emotional regulation) could be maintained after cessation of formal practice, which might be associated with a reduced likelihood of symptom relapse compared to abrupt medication withdrawal. However, it is crucial to note that robust, long-term (>1 year) RCT data directly comparing relapse rates between TC and pharmacotherapy are still limited, and these propositions require further longitudinal validation.

##### 3.1.3.4 Improvement of overall health

Beyond facilitating mental health, TC may also have beneficial effects on physical health. A body of research supports its potential advantages, notably regarding cardiovascular health, and some indicators of immune function, and sleep quality (Thelander and Ring, 2025; Li et al., 2001). Literature has determined that TC may benefit cardiovascular health, with studies showing associations with improved cardiac function, decreasing blood pressure, and facilitating cardiorespiratory endurance (Li et al., 2001; Ren et al., 2017). Moreover, preliminary research indicates that TC could contribute to overall health through mechanisms that may include modulation of immune-related parameters, with particular impacts uncovered notably in older adult populations (Li et al., 2001; Chen et al., 2016). About sleep, TC is considered positive in facilitating sleep quality. Systematic review has revealed that in the context of decreasing anxiety and stress levels, TC can help delay sleep disturbances, particularly among middle-aged and older adults (Ren et al., 2017). Unlike pharmacotherapy, which commonly targets notable symptoms, TC can employs a holistic method to modulation, aiding patients improve both their mental and physical health concurrently, hence elevating their overall health status (Li et al., 2001).

#### 3.1.4 Disadvantages of TC

These limitations collectively affirm that TC is best positioned as a complementary or maintenance therapy, rather than a primary intervention for acute psychiatric crises.

##### 3.1.4.1 Slower onset of action

The therapeutic benefits of TC typically require a period of sustained practice to become apparent, unlike the rapid onset of action seen with several medications (Zhu et al., 2010). This is primarily because TC, as a gentle mind–body exercise, works through gradually enhancing bodily coordination, balance, muscle strength, and improving cardiopulmonary function, immunity, and mental health (Li et al., 2001; Solloway et al., 2016). Literature has reported that while TC offers long-term advantages for patients with chronic conditions, such as arthritis and cardiovascular disease, its short-term relief effects are limited for those with acute symptoms or severe conditions (Hall et al., 2017). Therefore, TC is not suitable as a primary intervention for acute symptom crises but is better suited as a long-term adjunctive therapy in chronic disease management.

##### 3.1.4.2 Dependence on participant adherence and “dose” parameters

The effectiveness of TC is closely linked to participant adherence and the specific “dose” of practice—encompassing session duration, weekly frequency, and total intervention period (Yang et al., 2022). Significant health improvements, particularly in managing chronic conditions, require patients to engage in consistent and regular practice over weeks to months (Yang et al., 2022; Yang et al., 2015). This demand for a sustained time commitment and self-discipline can be a barrier. The literature commonly describes heterogeneous intervention protocols (e.g., 60-min sessions, two to three times per week, over 8–12 weeks), making it challenging to define a standardized optimal “dose” comparable to pharmacotherapy (Yang et al., 2015). For patients lacking self-management skills, time, or motivation, maintaining a regular TC practice at an effective dose can be challenging, which limits its recommendation in some populations (Yang et al., 2022).

##### 3.1.4.3 Lack of standardization

Although TC has a deep cultural background, issues regarding its standardization in clinical application indeed exist. A systematic review focusing on traditional Chinese exercise therapies noted that variations exist among different TC styles and teaching approaches, which can lead to inconsistent clinical outcomes (Jia et al., 2023). These individual differences are often linked to variations in instructor guidance (Jia et al., 2023). This lack of standardization extends to intervention “dosing,” contrasting with pharmacotherapy, which has strict dosing and usage guidelines providing more predictable effects in clinical practice (Jia et al., 2023).

### 3.2 Comparison between TC and cognitive behavioral therapy (CBT)

As a extensively utilized psychotherapy in clinical practice with significant article support, determining the advantages of CBT forms the basis for appreciating its position in the mental health range and function as a vital reference for the subsequent dimensional comparison with TC. Hence, this section centers on the advantages of CBT, assessing them from core dimensions, such as its targeted intervention impact, efficiency, and worth in skill cultivation, laying the groundwork for the integrative comparison that follows. Table 2 displays a comparative and integrated utilization analysis of TC and CBT in mental health intervention. CBT is a well-established, evidence-based first-line psychotherapy for a range of mood and anxiety disorders, with a vast body of research supporting its efficacy in symptom reduction and skill acquisition. The following comparison aims not to undermine this status, but to delineate how Tai Chi (TC), with its distinct mind–body approach, might offer complementary pathways or alternatives for specific patient subgroups and symptom domains.

#### 3.2.1 Advantages of cognitive behavioral therapy (CBT)

##### 3.2.1.1 High specificity

Cognitive Behavioral Therapy (CBT) positively addresses psychological issues according to aiding patients recognize and alter negative thought patterns and behavioral responses. Significant study displays CBT’s effectiveness in treating psychological disorders, such as anxiety, depression, and Post-Traumatic Stress Disorder (PTSD) (Öst et al., 2023; Bhattacharya et al., 2023). It can not only help patients in determining and challenging negative thoughts, but can also alters coping processes via behavioral modifications, hence facilitating mood and symptoms (Watkins et al., 2018). For PTSD, trauma-centered forms of CBT are notably suggested, assisting patients in processing trauma-associated emotions and memories (Nakao et al., 2021). Its wide applicability makes CBT one of the preferred treatment approaches for different mental health issues.

##### 3.2.1.2 Short-term effectiveness

CBT commonly generates impacts within a relatively short course, commonly 12 to 20 sessions. Via clear goal setting and step-by-step behavioral exercises, this therapy enables patients to learn cognitive and behavioral approaches for coping with life’s challenges in a short period. Literature has confirmed that setting explicit treatment goals not only improves therapeutic outcomes, but also aids patients sustain improvements after treatment concludes (Ramnerö and Jansson, 2016). In addition, the structured method of CBT supports active patient participation, and goal setting reinforces patients’ sense of control and engagement in the therapeutic mechansim (Ramnerö and Jansson, 2016).

##### 3.2.1.3 Skill-oriented approach

CBT centers not only on short-term symptom relief, but also on cultivating patients’ self-regulation skills. According to aiding individuals recognize and alter negative thought patterns and enhance self-awareness via behavioral change, CBT can promote the long-term utilization of these techniques, hence avoiding symptom relapse. It is noteworthy that these skills comprise self-monitoring, cognitive restructuring, and behavioral activation, which positively assist patients in controlling emotional and behavioral challenges in daily life (Nakao et al., 2021; Barton et al., 2023). For example, cognitive restructuring seeks to help patients recognize distorted negative thoughts and replace them with more adaptive ones (Nakao et al., 2021). Via these approaches, patients obtain not only short-term symptom alleviation, but also increased self-regulation abilities, hence decreasing the risk of relapse.

#### 3.2.2 Disadvantages of cognitive behavioral therapy (CBT)

##### 3.2.2.1 Needs cognitive engagement

Patient participation and capacity for self-reflection are critical in CBT. Nevertheless, implementing CBT can be challenging for patients with decreasing cognitive abilities or poor self-awareness, such as older adults or individuals in poor mental states (Mohlman and Gorman, 2005). Articles have displayed that cognitive functions, such as working memory, selective attention, and fluid intelligence may decrease with age, impacting information processing and self-reflection capabilities in the context of CBT (Evans, 2007; Laidlaw et al., 2003). For these patients, notably those with vital cognitive impairment, severe depression, or anxiety, conventional CBT may have restricted effectiveness (Manard et al., 2014). Adaptations may be necessary, such as outlining information more slowly, improving repetition and summarization, or utilizing numerous sensory modalities (e.g., visual, auditory).

##### 3.2.2.2 Potential for emotional fluctuations in the context of treatment

The CBT mechanism commonly needs patients to confront and process negative emotions and traumatic experiences, notably trauma-associated emotional responses (Alpert et al., 2021). Short-term emotional fluctuations and discomfort are relatively general in the context of this mechanism, notably in the initial stages of treatment or when handling sensitive trauma (Houben et al., 2015). For patients with severe trauma or high emotional sensitivity, the first emotional reactions can be notably intense, possibly causing emotional overwhelm or vital distress (Watkins et al., 2018; Murray et al., 2022). It is noteworthy that these fluctuations are part of the therapeutic mechanism; in the context of gradually experiencing these emotions, patients can ultimately decrease the influence of negative feelings and regain management over their lives.

##### 3.2.2.3 Dependency on therapist skill

It is worth noting that literature has built that the effectiveness of CBT counts on the therapist’s experience and skill level. The therapist’s proficiency, experience, and mastery of CBT techniques directly impact treatment outcomes. A skilled therapist can not only guide problem identification, but can also promote positive cognitive modification. Faced therapists can respond more precisely to patient requirements, improving effectiveness, whereas inadequate guidance or reduction of skill can vitallydecrease therapeutic outcomes (Gkintoni et al., 2025).

#### 3.2.3 Comparative advantages of TC versus CBT

##### 3.2.3.1 Emphasis on somatic awareness

In comparing TC and CBT, TC places greater emphasis on bodily awareness and modulation. Via delay physical movements, breath regulation, and mindfulness practice, TC positively delays physiological symptoms associated with psychological issues, such as anxiety and depression, such as muscle tension and wide breathing (Kanani et al., 2025; Qu et al., 2024). This mind–body practice compensates for a relative reduction of center on physiological relaxation and modulation in CBT, which mainly targets cognitive modification (Qu et al., 2024). Literature has reported that integrating mindfulness with somatic modulation in TC not only promotes mental health, but also decreases symptoms of anxiety and depression under the backdrop of impacting the autonomic nervous system (Kanani et al., 2025). In comparison to CBT, the movement perspective of TC can activate the parasympathetic nervous system, aiding individuals sustain better physiological balance in the context of stress (Kuang et al., 2024). Hence, integrating TC with CBT can offer a more integrative treatment method for anxiety and depression, handling both thought patterns and physical symptoms.

##### 3.2.3.2 Non-verbal modulatory approach

Literature can encourage the effectiveness of both CBT and TC in emotion regulation and trauma treatment. CBT mainly relies on language and cognitive analysis, aiding patients delay emotional issues under the backdrop of determining and changing negative thought patterns (Fordham et al., 2018). Conversely, TC, as a non-verbal physical practice, promotes emotion regulation via slow, rhythmic movements, making it notably suitable for patients less adept at verbal emotional expression (Chen et al., 2021; Zhong et al., 2021). For some patients with trauma histories, particularly those who experience significant distress upon verbal recounting of traumatic events (Gjerstad et al., 2024; Niles et al., 2022). Literature has indicated TC helps modulate trauma-associated physical tension and delays symptoms, such as anxiety, hence preventing psychological discomfort associated with verbally recounting traumatic experiences (Laskosky et al., 2023). This method is notably suitable for trauma patients who find verbal therapy challenging.

##### 3.2.3.3 Mindfulness and relaxation impacts

TC helps patients obtain a mindful state via slow movements and focused breathing, sharing similarities with Mindfulness-Based Cognitive Therapy (MBCT) utilized in CBT (Gkintoni et al., 2025). This mindfulness practice can help decrease rumination on negative emotions and promote self-awareness and emotion regulation skills (Rodrigues et al., 2024; Chan et al., 2020). In addition, Literature has confirmed that TC, such as MBCT, can enhance self-efficacy, which is critical for facilitating emotion regulation and mental health (Rodrigues et al., 2024; Chan et al., 2020).

#### 3.2.4 Potential advantages of combined use

##### 3.2.4.1 Improving overall effectiveness

Articles on integrating CBT and TC have reported that their integration can yield vital influences on both psychological and physiological levels (Irwin et al., 2015; Irwin et al., 2014). CBT mainly aids patients cope with stress and anxiety in the context of altering thought patterns and behavioral responses, while TC improves internal balance and decreases physical tension and stress in the context of influencing the body and emotions (Nakao et al., 2021; Wang et al., 2014). This integrated method has been reported to decrease inflammation levels and cause enhanced immune function, notably in patients with anxiety disorders, insomnia, and other chronic conditions (Liu et al., 2018). Moreover, studies have reported that the integration of CBT and TC can positively promote balance and decrease the fear of falling in older adults, with effective impacts maintained over the long term (6 to 12 months) (Liu et al., 2018). Combining these two interventions can promote integrative rehabilitation, both psychologically and physiologically.

##### 3.2.4.2 Decreasing emotional fluctuations

Patients may face emotional fluctuations in the context of CBT under the backdrop of confronting negative emotions (Laicher et al., 2025). Literature has indicated that incorporating the mindfulness practice of TC can positively help patients modulate emotions, notably stress responses associated with anxiety and depression (Wang et al., 2014; Shen et al., 2023). For instance, Literature has uncovered that TC, as a mind–body exercise stressing physical relaxation and mindful focus, can vitally decrease symptoms of depression and anxiety (Kanani et al., 2025; Qu et al., 2024). Besides, TC can help patients sustain calm in the context of treatment under the backdrop of improving their awareness of the present moment, avoiding excessive emotional swings caused by negative feelings (Zeng et al., 2023). This somatic method to relaxation complements the psychological intervention of CBT, making emotion control in the context of treatment more positive.

##### 3.2.4.3 Facilitating self-regulation capacity

Integrating CBT and TC can help patients better control stress and emotions on both psychological and physiological levels. Study displays that CBT improves emotion regulation in the context of training patients to recognize negative thoughts and alter behaviors (Goldin et al., 2014; Forkmann et al., 2014). TC, as a mind–body practice, promotes self-awareness and relaxation techniques via its feature delay movements and breath control (Zhang et al., 2019; Wang et al., 2023). Notably Literature has uncovered that TC can vitally promote emotion regulation and promote self-regulation capacity, which is notably positive in decreasing negative emotions, such as anxiety and depression (Wang et al., 2023). It is noteworthy that these findings imply that integrating CBT with TC not only handles emotional issues from a cognitive standpoint, but also improves self-regulation of stress responses via physical practice, providing a more integrative intervention approach at the mind–body level.

##### 3.2.4.4 Adapting to various patient types

For several patients, cognitive modification (as in CBT) might be more negative, notably initially if they lose sufficient self-reflection capacity (Perivoliotis et al., 2010). At such times, TC, as a mind–body exercise, can offer a more direct intervention pathway. Study has confirmed that in the context of combining physical movement, breath control, and mental focus, TC can promote cognitive function and displays several impact in the early prevention and mitigation of cognitive impairment (Chen et al., 2021; Jasim et al., 2023). Conversely, for patients more adept at controlling emotions via cognitive analysis, CBT can offer a systematic cognitive training approach to cope with psychological distress under the backdrop of restructuring thought patterns (Kazantzis et al., 2018; Monachesi et al., 2023). Utilizing both modalities in integration allows for flexible modification of the intervention plan according to individual patient requirements, hence improving treatment adaptability and effectiveness (Chen et al., 2021). This individualized intervention method aims better carry out the requirements of patients with various types of psychological or cognitive issues.

### 3.3 Comparison of TC with other mind–body exercises: a focus on clinical applicability

When considering TC alongside other prevalent mind–body exercises such as Yoga and Qigong, the key question for clinical practice is not which is superior, but how their distinct profiles suit different patient needs, capabilities, and treatment contexts. All three practices share the common goal of promoting mind–body integration and have demonstrated benefits for mental health (Chan et al., 2013; Liu et al., 2020; Liu et al., 2021). However, critical differences in movement characteristics, intensity, learning demands, and cultural-therapeutic framing have direct implications for their clinical application.

#### 3.3.1 Comparative analysis of Core distinctions

A concise, clinically-oriented comparison is presented in Table 3. The analysis highlights several pivotal distinctions. Yoga often emphasizes static postures (asanas) and flexibility, with intensity ranging widely from gentle (e.g., Yin Yoga) to physically demanding (e.g., Power Yoga). This variety allows for tailoring but requires careful selection for frail populations. In contrast, TC is characterized by continuous, flowing movements and dynamic balance training at a consistently low-to-moderate intensity, making it inherently safer and more accessible for individuals with limited mobility or chronic conditions (Khajuria et al., 2023; Wehner et al., 2021). Qigong typically involves even simpler, repetitive movements or static poses with minimal physical exertion, representing the lowest intensity option (Wehner et al., 2021; Chen et al., 2022). The complexity of movement sequences differs significantly. TC forms (e.g., the 24-form) are interconnected and require learning coordinated transitions, presenting a moderate learning curve (Hu et al., 2021). Yoga’s learning demand varies with style but often involves mastering specific alignments. Qigong, with its simple, repetitive movements, has the lowest barrier to entry, allowing beginners to engage quickly with basic techniques (Wehner et al., 2021; Chen et al., 2022). This difference in complexity directly influences initial adherence, the need for qualified instruction, and the time to independent practice. While all practices incorporate breath and awareness, their hypothesized primary mechanisms differ in emphasis. Yoga often strongly links physical posture, flexibility, and breath control (pranayama) to psychological state (Khajuria et al., 2023; Seshadri et al., 2020). TC’s core is the integration of continuous movement with mindful awareness and balance control, proposed to enhance parasympathetic nervous system activity and body–mind coordination (Qu et al., 2024; Zou et al., 2018). Qigong most explicitly centers on the cultivation and regulation of “Qi” (vital energy) through breath and gentle intent, often viewed as a form of “moving meditation” for deep relaxation (Yeung et al., 2018; Wang et al., 2014). These emphases, while overlapping, can guide their selection for patients with different symptom presentations (e.g., somatic tension vs. cognitive agitation vs. pervasive fatigue).

#### 3.3.2 Implications for clinical decision-making

These distinctions translate into pragmatic considerations for intervention planning. For physically frail, elderly, or acutely stressed patients: Qigong or very gentle Yoga may be optimal starting points due to low physical and cognitive demand. TC is also highly suitable, particularly if fall prevention and dynamic balance are secondary goals. For patients seeking to improve flexibility, strength, or tolerating higher intensity: Styles of Yoga designed for these outcomes are more appropriate. For patients needing a structured, rhythmical practice that emphasizes continuity and present-moment focus without spiritual/cultural overlay: TC’s standardized forms (like the 24-form) offer a clear framework. For integration into busy lifestyles or as a preliminary skill-building intervention: The simplicity of Qigong facilitates home practice and may improve readiness for more complex interventions like TC or CBT.

#### 3.3.3 Potential for combined practice

Integrating elements from these practices can address multidimensional needs. For example, a regimen could combine Yoga for flexibility, TC for balance and continuity, and Qigong for deep relaxation, tailored to an individual’s weekly schedule and energy levels. Such an integrated approach may enhance overall adherence by providing variety and targeting multiple wellness domains (Wang et al., 2017). Future research should explore the feasibility and efficacy of such personalized, multimodal mind–body prescriptions.

## 4 Clinical implementation challenges and evidence limitations

Although this review reveals the potential of TC as an auxiliary intervention for mental health, successfully integrating it into routine clinical practice and public health systems still faces a series of clear challenges. A candid examination of these challenges is crucial for interpreting existing evidence, guiding future research, and clinical practice.

### 4.1 Security, reporting standards, and feasibility challenges

TC is generally considered a safe low-intensity exercise. However, this general understanding may lead to insufficient systematic reporting of adverse events (AEs) in research. Many clinical trials lack standardized collection and reporting of AEs, making it difficult to comprehensively assess their risk in special populations such as those with severe osteoporosis, uncontrolled hypertension, and poor balance function. Future research must adopt international standards such as CONSORT to actively monitor and transparently report AEs, in order to establish more reliable safety records. In terms of feasibility, the key challenges lie in long-term compliance and accessibility in the real world. Compared to taking a medication or attending a treatment session, adhering to TC exercises multiple times a week for several months places higher demands on patients’ motivation, time management, and self-management abilities. Research often reports a decrease in practice frequency over time (Yang et al., 2022; Yang et al., 2015). In addition, qualified TC mentors are not readily available, and course fees, venue limitations, and cultural acceptance may also be obstacles. Digital health technologies, such as online video courses and VR, have the potential to partially address accessibility issues, but their effectiveness and applicability to different populations, such as elderly people with low digital literacy, still need to be evaluated.

### 4.2 Heterogeneity and standardization dilemma of intervention plans

A core bottleneck in this field is the significant heterogeneity of intervention plans. There are significant differences in TC style (such as Yang style, Chen style), single session duration (30 vs. 60 min), weekly frequency, total intervention period (8 vs. 24 weeks), and teaching focus among different studies (Jia et al., 2023; Hu et al., 2021). This inconsistency in “dosage” makes it difficult to directly compare studies, hinders the clear establishment of a “dose–response” relationship, and poses difficulties for the development of clinical guidelines and the replication of results. Despite the existence of relatively standardized routines such as “simplified 24 TC”, achieving complete standardization in clinical research remains difficult. Differences in teaching quality and the integration of personal styles of instructors may become confounding variables for intervention effectiveness. Future effectiveness research needs to focus on developing and reporting detailed and reproducible intervention manuals that clearly define core motor elements, respiratory coordination, mind guidance, and allowable adjustment ranges. This is a key step toward advancing this field toward evidence-based medicine.

### 4.3 Critical synthesis of limitations of existing evidence

When synthesizing existing literature, one must carefully consider its inherent limitations many positive findings come from randomized controlled trials with limited sample sizes and short follow-up times. The evidence for the long-term (>1 year) recurrence prevention effect of TC is more based on theoretical inference and observational studies, rather than conclusive long-term RCT data. Meanwhile, there are conflicting findings in the literature, such as insignificant effects in certain subgroup analyses, which may be related to the heterogeneity of interventions, sample characteristics, or differences in measurement tools mentioned above. Like many fields of complementary medicine, TC research may have publication bias, where studies with positive results are more likely to be published. This may lead to an overestimation of the effect size. In addition, the clinical and methodological heterogeneity included in the study (such as baseline conditions of participants and control group settings) is high, which requires us to be extra careful when interpreting comprehensively and avoid making overly uniform conclusions.

### 4.4 Cautious implications for clinical guidelines

Given the challenges and limitations mentioned above, current evidence is insufficient to support TC as a monotherapy alternative to drug therapy or CBT for acute or severe mental disorders. However, there are sufficient reasons to consider it as a valuable evidence-based complementary and integrated therapy. The inspiration for clinical guidelines should be encouraging rather than directive: guidelines can recognize TC as an optional supplementary intervention for patients with mild to moderate depression, anxiety, or comorbidities of chronic physical diseases. When recommending, emphasis should be placed on its auxiliary positioning, and it is recommended to start with low-intensity, short cycle plans, with particular attention to the patient’s personal interests and physical tolerance, in order to improve compliance. Clinical decision-making should be individualized, taking into account the patient’s stage of illness, physical function, personal preferences, and accessibility of resources. By facing up to these challenges and limitations, the field can move forward more solidly. Future research should focus on conducting high-quality, standardized and practical clinical trials, and exploring in depth how to overcome implementation barriers, ultimately enabling more patients to benefit from this ancient physical and mental practice.

## 5 Conclusion and prospects

### 5.1 Conclusion

Our review delve into comparing the roles and features of TC, pharmacotherapy, CBT, and other conventional exercises (e.g., Yoga, Qigong) in mental health interventions. By synthesizing recent articles, evidence suggests that TC, as a multi-level and multi-target mind–body exercise, may play a valuable role in mental health improvement and disease intervention. In summary, the core value of TC appears to lie in its “mind–body integration” intervention model. In comparing TC to pharmacotherapy, we have employed cautious language to reflect the heterogeneity of the evidence. Claims regarding TC’s superior long-term maintenance and relapse prevention are promising but are currently supported more by mechanistic reasoning and medium-term studies than by definitive long-term randomized data. This distinction underscores the need for more longitudinal comparative effectiveness trials. Although TC has a relatively slower onset of action, its benefits include the absence of drug-associated side impacts, the ability to generate maintained long-term impacts, and potential improvements in overall health. It is notably suitable for chronic disease control, older adult populations, and patients intolerant to medications. In comparison to psychotherapies, such as CBT, which center on cognitive restructuring, TC may offer a non-verbal, body-awareness-based intervention pathway. It has been associated with positive influences on the autonomic nervous system and the alleviation of physiological symptoms of anxiety and depression, which can be notably advangeous for individuals who are less adept at verbal expression or those with vital somatic symptoms. In comparison to other mind–body practices, such as Yoga and Qigong, TC is distinctive in its movement continuity, balance training, and cultural-philosophical foundations. Its lower intensity and slower pace also grant it broader applicability and safety. It is more important that these article significant encourages an combined intervention approach. TC is not intended to replace pharmacotherapy or CBT, but to complement them. For example, for severely ill patients in the acute phase, a model of “pharmacotherapy for wide symptom management, with TC for long-term rehabilitation and maintenance therapy” can be utilized. For patients experiencing CBT, TC could serve as an adjunct, potentially reinforcing the outcomes of cognitive modification via physical practice and helping to mitigate emotional fluctuations during treatment. This diversified method, integrating medication, psychology, and exercise, represents a promising approach to exploring more holistic and personalized health management strategies.

### 5.2 Prospects

Looking ahead, the following perspectives warrant further in-depth research: The current understanding of the neurobiological processes underlying TC’s effects (e.g., influences on specific neurotransmitter systems, brain network function, neuroplasticity) remains preliminary. Future studies need to utilize advanced technologies, such as fMRI, EEG, and biomarker detection to conduct more rigorously designed mechanistic studies, offering a solid scientific foundation for the clinical application of TC. The existence of numerous TC styles and variations in teaching poses challenges for the reproducibility of clinical research and its broader dissemination. Future studies should focus on developing standardized, quantifiable TC intervention protocols. Crucially, research must establish clearer dose–response relationships, systematically investigating how variables such as session length (e.g., 30 vs. 60 min), frequency (e.g., daily vs. bi-weekly), total duration (e.g., 8 vs. 24 weeks), and movement complexity influence specific mental health outcomes. Building on this, investigating personalized protocols (including personalized “dosing”) for various diseases and populations (e.g., varying ages, comorbidities) should be pursued to maximize effectiveness and adherence. More large-sample, long-term follow-up RCTs are required to determine the value of TC in relapse prevention and improving long-term prognosis. These trials should meticulously report and control for intervention “dose” parameters. Concurrently, conducting health economic evaluations to explore its potential for reducing healthcare costs and improving cost-effectiveness within healthcare systems, both in China and worldwide, would significantly advance its integration into mainstream medicine. Investigating the adaptability and effectiveness of TC in various cultural contexts is key to promoting its global dissemination. In addition, with the rise of digital health technologies, developing TC intervention programs delivered via video guidance, virtual reality (VR), or wearable devices holds the potential to address time and geographical constraints, thereby improving patient accessibility and adherence. These digital platforms also offer novel opportunities to precisely monitor practice “dose” (frequency, duration) and provide adaptive, personalized training regimens. Future high-quality RCTs should not only evaluate TC alone but, more critically, test well-defined adjunctive protocols (e.g., TC + standard pharmacotherapy vs. standard care alone) across different clinical phases and specific patient subgroups to establish evidence-based integration guidelines.
